# Inequalities in use of total hip arthroplasty for hip fracture: population based study

**DOI:** 10.1136/bmj.i2021

**Published:** 2016-04-28

**Authors:** Daniel C Perry, David Metcalfe, Xavier L Griffin, Matthew L Costa

**Affiliations:** 1 Institute of Translational Medicine, University of Liverpool, Liverpool, L12 2AP, UK; 2 Centre for Surgery and Public Health, Harvard Medical School, Boston, MA 02115, USA; 3 Nuffield Department of Orthopaedics, Rheumatology, and Musculoskeletal Sciences, University of Oxford, The Kadoorie Centre, John Radcliffe Hospital, Oxford OX3 9DU, UK

## Abstract

**Objectives** To determine whether the use of total hip arthroplasty (THA) among individuals with a displaced intracapsular fracture of the femoral neck is based on national guidelines or if there are systematic inequalities.

**Design** Observational cohort study using the National Hip Fracture Database (NHFD).

**Setting** All hospitals that treat adults with hip fractures in England, Wales, and Northern Ireland.

**Participants** Patients within the national database (all aged ≥60) who received operative treatment for a non-pathological displaced intracapsular hip fracture from 1 July 2011 to 31 April 2015.

**Main outcome measures** Provision of THA to patients considered eligible under criteria published by the National Institute for Health and Care Excellence (NICE).

**Results** 114 119 patients with hip fracture were included, 11 683 (10.2%) of whom underwent THA. Of those who satisfied the NICE criteria, 32% (6780)****received a THA. Of patients who underwent THA, 42% (4903) did not satisfy the NICE criteria. A recursive partitioning algorithm found that the NICE eligibility criteria did not optimally explain which patients underwent THA. A model with superior explanatory power drew distinctions that are not supported by NICE, which were an age cut off at 76 and a different ambulation cut off. Among patients who satisfied the NICE eligibility, the use of THA was less likely with higher age (odds ratio 0.88, 95% confidence interval 0.87 to 0.88), worsening abbreviated mental test scores (0.49 (0.41 to 0.58) for normal cognition *v* borderline cognitive impairment)), worsening American Society of Anesthesiologists score (0.74, 0.66 to 0.84), male sex (0.85, 0.77 to 0.93), worsening ambulatory status (0.32, 0.28 to 0.35 for walking with a stick *v* independent ambulation), and fifths of worsening socioeconomic area deprivation (0.76 (0.66 to 0.88) for least *v* most deprived fifth). Patients receiving treatment during the working week were more likely to receive THA than at the weekend (0.90, 0.83 to 0.98).

**Conclusions** There are wide disparities in the use of THA among individuals with hip fractures, and compliance with NICE guidance is poor. Patients with higher levels of socioeconomic deprivation and those who require surgery at the weekend were less likely to receive THA. Inconsistent compliance with NICE recommendations means that the optimal treatment for older adults with hip fractures can depend on where and when they present to hospital.

## Introduction

There are over 70 000 hip fractures in the United Kingdom every year, with a combined health and social cost of £2bn (€2.5bn, $2.8bn).^1^ Demographic projections estimate that the annual incidence will increase to over 100 000 by 2020.^2^ Mortality is high, with 8.5% of patients dying within 30 days after hip fracture.^3^

Several initiatives have been credited with improving outcomes in the UK.^3^ In 2004 the British Orthopaedic Association (BOA) and the British Geriatrics Society (BGS) established the National Hip Fracture Database (NHFD), with the aim of improving outcomes of hip fracture through continuous national clinical audit.^4^ The national database was supported by combined BOA/BGS clinical guidance^5^ and later by the best practice tariff for hip fracture, which rewards NHS organisations for meeting defined quality standards, including surgery within 36 hours after arrival at hospital.^6^ These initiatives have been associated with improved outcomes, including a fall in 30 day mortality from 10.9% in 2007 to 8.5% in 2011.^3^

Displaced intracapsular hip fractures are at high risk of painful non-union and so the recommended treatment is either hemiarthroplasty or total hip arthroplasty (THA).^7^ ^8^ ^9^ In hemiarthroplasty, the femoral head is replaced; in THA, both the femoral head and acetabulum are replaced. Although the risk-benefit profiles vary between these two operations, it has been shown that patients who undergo THA have better function and less need for revision surgery.^7^ ^9^ ^10^ ^11^ In June 2011, the National Institute for Health and Care Excellence (NICE) recommended that THA should be offered to patients with a displaced intracapsular hip fracture who are “(a) able to walk independently out of doors with no more than the use of a stick (b) not cognitively impaired and (c) medically fit for anaesthesia and the procedure.”^8^ The provision of THA is not explicitly included as a quality indicator within the NHFD and so the extent to which surgeons comply with this guideline is unknown.

From clinical experience, we hypothesised that there were inequalities in use of THA between hospitals. We identified whether the use of THA is based on factors that are consistent with national recommendations or if systematic inequalities exist with regards to the use of THA for hip fracture.

## Methods

We carried out an observational study using data collected by the NHFD national clinical audit project. The study protocol was approved by the Healthcare Quality Improvement Partnership (HQIP) before data release, but research ethics committee approval was not sought for secondary analysis of administrative data in line with Governance Arrangements for Research Ethics Committee (GAfREC) guidelines.^12^

### Data source

The NHFD is commissioned by the Healthcare Quality Improvement Partnership and managed by the Royal College of Physicians as part of the Falls and Fragility Fracture Audit Programme (FFFAP). It captures over 95% of hip fractures treated in England, Wales, Northern Ireland, and the Channel Islands. Data include patients’ characteristics, fracture pattern, surgical interventions, and outcomes. These details are typically collected by specialist nurses within each hospital who provide continuity of care to patients with hip fractures and manage submissions to the NHFD. Data from patients aged under 60 are not captured within the database.

### Inclusion criteria

This study included all patients aged ≥60 who presented to hospital from 1 July 2011 to 31 April 2015 with a displaced intracapsular hip fracture. We chose 1 July 2011 as one month after publication of NICE Clinical Guideline 124.^8^ Patients were excluded if their fracture was coded as “pathological” as this could represent a heterogeneous group that includes patients with disseminated cancer.

### Variables and outcomes

Data cleaning involved several steps. Two patients had ages recorded as >115 (both >1000), which we recoded to exclude this variable. In 27 (0.01%) cases, the score of the abbreviated mental test (AMTS) was not recorded as an integer and so scores were rounded to the nearest integer. On 1 April 2014 the NHFD data collection tool was updated to record mobility differently within the revised database. Earlier data were therefore mapped onto the new version by using the algorithm shown in appendix 1. In the event of hospital trust reconfiguration (closure/merger), we used the hospital code at the time of data entry. As a consequence, some hospitals contributed data for only a few months before reconfiguration.

Variables extracted from the NHFD were age (whole years), sex, lower layer super output area (LSOA), date of admission, treating hospital, pre-morbid mobility, American Society of Anesthesiologists (ASA) classification score for physical status, and score on the abbreviated mental test. The physical status score ranges between 1 (healthy patient) and 5 (moribund patient not expected to survive for 24 hours with or without surgery). The abbreviated mental test is a test of 10 questions (such as “what is your age?”), which gives a score from 0 (zero answers correct) to 10 (all correct).

Deprivation scores for patients living in England were determined with the index of multiple deprivation, 2007. These scores reflect deprivation related to income, health and disability, employment, barriers to housing and services, living environment, education, and crime.^13^ Scores were generated from lower layer super output areas, which were then categorised into fifths of deprivation based on the population of the UK.

Day of the week was determined from the date of admission. In the UK, surgery for hip fracture usually takes place on the next available trauma operating list, which for most patients in the NHFD (≥65%) is the day after admission. “Weekend” surgery was therefore identified by admission on a Friday or Saturday.

Hospital case volume was analysed by 10ths and defined by the number of people with displaced intracapsular fracture admitted to each centre over the study period.

Date of surgery was analysed as seven periods of six months (1 July 2011 to 31 December 2015) and one period of four months (1 Jan 2015 to 31 April 2015).

### Statistical analysis

We determined compliance with guidelines with a decision tree ordered to mirror the NICE recommendations—that is, based on mobility (mobile outdoors with or without the use of a stick), cognition (defined as mental test score ≥8), and fitness for anaesthesia (defined as physical status score 1 or 2). Although the cut offs used for these two scores are not expressly published as part of the guideline, they have been used by NICE to monitor compliance with the guideline.^14^ A mental test score <8 has previously been shown to identify cognitive impairment^15^ and has been adopted as a threshold by the Royal College of Physicians of London.^16^ We determined the extent to which the NICE algorithm explained practice—that is, those individuals correctly classified as a percentage of the total.

We used recursive partitioning to determine the optimal decision tree that explains current practice—that is, to illustrate how the guidelines are being interpreted. Recursive partitioning is a statistical technique for multivariable analysis that models how variables are best organised to predict a given outcome (such as THA). Decision trees are built by identifying a variable that best splits the data into two groups. The partitioning process defines a cut off (split) for continuous or ordinal variables to enable the decision tree to correctly classify the maximum members of the population. Categorical variables are similarly grouped to build a tree with the least error. This process is then applied separately to each subgroup and continues recursively until either a maximum number of steps are reached or no further improvement is possible.^17^

We undertook recursive partitioning using the “rpart” function in R. The tree was built with 10-fold cross validation and a negative complexity parameter to ensure that the maximum tree was built. Predictors included in the model were age, sex, mobility, cognition (AMTS), physical status (ASA score), fifth of index of multiple deprivation, and day of the week of admission. The tree was pruned with the complexity (“cp”) function of the smallest tree within one standard error of the best functioning tree—that is, the tree with the smallest xerror, which was confirmed graphically. We also used a pragmatic approach to consider the tree complexity and efficiency related to clinical practice.

Individuals who fulfilled the NICE criteria were further analysed to explore factors associated with undergoing THA. We constructed a recursive partitioning decision tree to differentiate between THA and no THA in this subgroup. The treating hospital was included as a factor variable, which allowed the partitioning algorithm to select optimal cut off points for best fit within the model.

We constructed a mixed effects logistic regression model to explore factors associated with the use of THA among patients who fulfilled the NICE criteria. Age, sex, date of surgery, cognition, and physical status were included as continuous predictors; and fifth of index of multiple deprivation and weekend surgery as categorical predictors. Weekend admission was then substituted for day of the week to explore this predictor further in a second analysis. Hospital case volume was included as a centre level fixed effect and the unique hospital identifier as a centre level random effect. We applied the same analysis to patients who did not fulfil the NICE criteria for THA to determine factors predictive of receiving a THA in this group.

Statistical analyses were performed with R and Stata version14.0. P<0.05 was adopted as the threshold for significance.

### Patient involvement

No patients were involved in setting the research question or the outcome measures, nor were they involved in developing plans for design or implementation of the study. No patients were asked to advise on interpretation or writing up of results. There are no plans to disseminate the results of the research to study participants or the relevant patient community.

## Results

In the 46 month period between 1 July 2011 and 31 April 2015, the NHFD recorded 248 013 patients with hip fracture. Of these, 114 119 satisfied the study criteria with a non-pathological displaced intracapsular hip fracture. Though 21 193 patients satisfied the NICE criteria to receive a THA (fig 1), only 11 683 within the NHFD underwent THA. Among these 11 683 patients, 4903 did not fulfil the NICE criteria.

**Figure f1:**
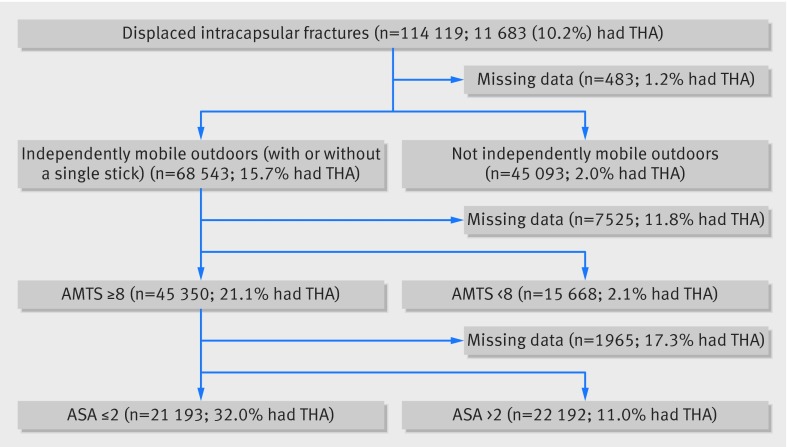
**Fig 1** Decision tree for total hip arthroplasty (THA) in displaced intracapsular fractures as per NICE guidelines. AMTS=abbreviated mental test score, ASA=American Society of Anesthesiologists score

The recursive partitioning algorithm identified 10 terminal nodes (nine splits) as the most predictive model, although this offered little improvement over five terminal nodes (four splits) (fig 2). The variable with the greatest importance was patient age, with a cut off age of 77 defining the initial split (fig 3). The mobility split occurred between patients who ambulate independently and those who required the use of a stick. The other important predictive variables were those recommended by NICE, with splits occurring as predicted at ASA ≥3 for physical status and AMTS ≥8 for cognition. With the decision tree, the explained practice across the dataset improved from 82.7% (NICE guidelines) to 90.4% (recursive model).

**Figure f2:**
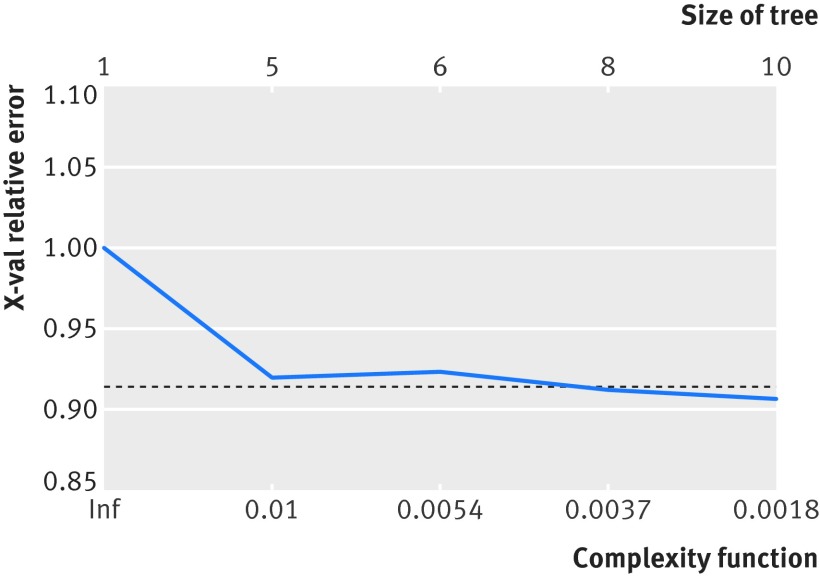
**Fig 2** Graph illustrating limited improvement in model using optimal tree size of 10 terminal nodes (lowest error), and more pragmatic tree with five nodes

**Figure f3:**
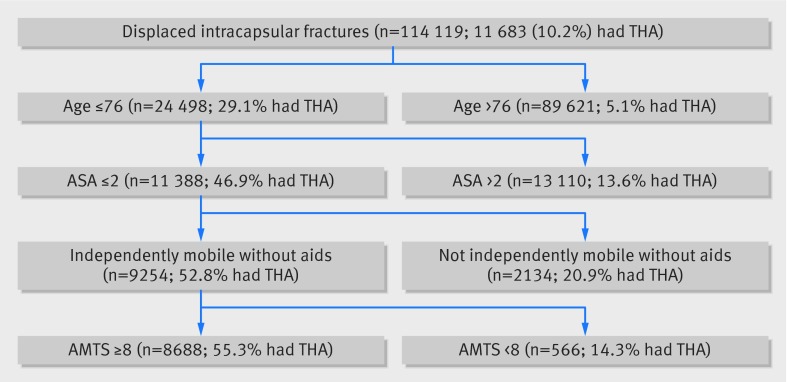
**Fig 3** Decision tree for total hip arthroplasty (THA) in displaced intracapsular fractures using recursive partitioning algorithm

Among the 21 193 patients fulfilling the NICE eligibility criteria, the recursive partitioning algorithm identified 20 terminal nodes (19 splits) to be the most efficient, although after three splits (four terminal nodes), the complexity of the tree increased markedly with little associated gain in efficiency (fig 4). Again age was the most significant predictor, with aged 79 identifying the splitting point (fig 5). For patients aged ≥79, the treating hospital was the next most important predictor (see appendix 2 for further details), followed by mobility (with or without the use of a stick). Hospital variation among individuals fulfilling the NICE guidelines was considerable (fig 6). Of the variation in practice, 77% could be explained using this recursive partitioning algorithm, compared with 32% by NICE guidelines alone.

**Figure f4:**
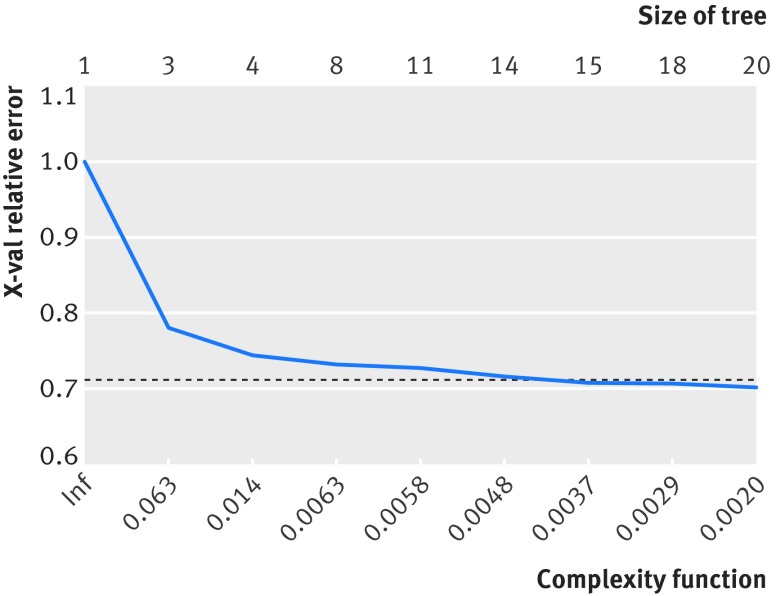
**Fig 4** Graph illustrating limited improvement in model using optimal tree size of 20 terminal nodes and simplified tree with four nodes

**Figure f5:**
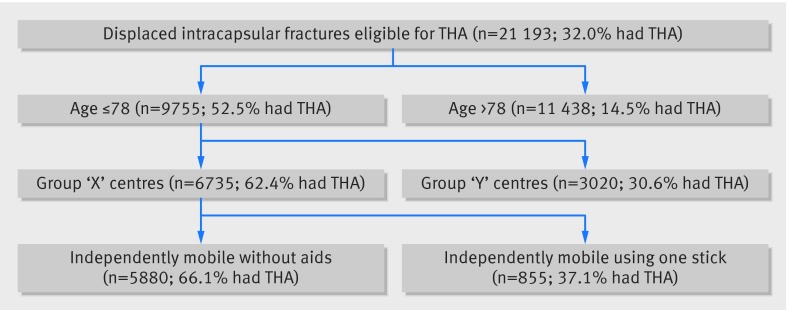
**Fig 5** Decision tree using recursive partitioning algorithm to indicate important predictors for total hip arthroplasty (THA) among individuals fulfilling NICE criteria for consideration of THA

**Figure f6:**
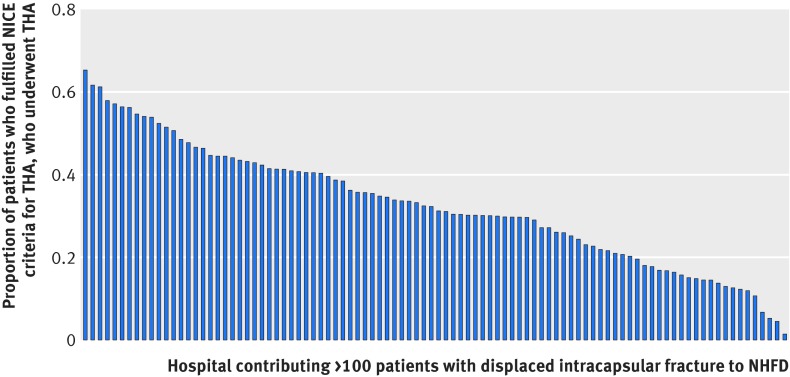
**Fig 6** Variation in number of total hip arthroplasty (THA) performed within each hospital as proportion of total number of individuals fulfilling NICE guidelines. Only hospitals that contributed >100 NICE eligible patients are included to minimise spurious data (n=96). Each bar represents one hospital

Date of surgery showed that there was a progressive increase in the provision of THA for eligible individuals over the study period (table 1).

**Table 1 tbl1:** Proportion of eligible patients who underwent total hip arthroplasty (THA) by time period

Period	Individuals undergoing THA/individuals fulfilling NICE criteria for THA (%)
1 July 2011-31 Dec 2011 (6 months)	453/2020 (22)
1 Jan 2012-30th June 2012 (6 months)	649/2409 (27)
1 July 2012-31 Dec 2012 (6 months)	804/2703 (30)
1 Jan 2013-30th June 2013 (6 months)	942/3041 (31)
1 July 2013-31 Dec 2013 (6 months)	1007/3099 (32)
1 Jan 2014-30th June 2014 (6 months)	1104/3077 (36)
1 July 2014-31 Dec 2014 (6 months)	1160/3094 (37)
1 Jan 2015-30th April 2015 (4 months)	661/1750 (38)

The logistic regression model (table 2) showed that 10ths of hospital volume did not affect THA (odds ratio 1.02, 95% confidence interval 0.97 to 1.08). Increasing age (0.88, 0.87 to 0.88), poorer cognition (AMTS) (0.49 (0.41 to 0.58) for 1.0 (ref) *v* borderline for cognitive impairment), and worsening physical status (ASA score) (0.74, 0.66 to 0.84), however, were associated with fewer procedures, as was male sex (0.85, 0.77 to 0.93). Admissions for surgery during the working week had the highest odds for receipt of THA (weekend admission 0.90, 0.83 to 0.98). There was a stepwise decrease in the odds of receiving THA with worsening area deprivation, such that the most deprived fifth had the fewest procedures (0.76, 0.66 to 0.88).

**Table 2 tbl2:** Mixed effects logistic model showing odds of receiving total hip arthroplasty (THA) among those deemed eligible by NICE guidelines

Variable	OR (95% CI)	P value
Age (for each increasing year of life)	0.88 (0.87 to 0.88)	<0.001
AMTS:		
10 (maximum correct answers)	1.0 (ref)	—
9	0.69 (0.62 to 0.77)	<0.001
8 (borderline for cognitive impairment)	0.49 (0.41 to 0.58)	<0.001
ASA score:		
1 (healthy person)	1.0 (ref)	—
2 (mild systemic disease)	0.74 (0.66 to 0.84)	<0.001
Mobility:		
Walks independently without aids	1.0 (ref)	—
Walks with aid of one stick	0.32 (0.28 to 0.35)	<0.001
Sex:		
Female	1.0 (ref)	—
Male	0.85 (0.77 to 0.93)	0.002
Day of admission:		
Saturday	0.86 (0.75 to 0.99)	0.03
Sunday	0.88 (0.76 to 1.12)	0.09
Monday	0.93 (0.81 to 1.07)	0.32
Tuesday	0.94 (0.82 to 1.08)	0.41
Wednesday	1.00 (ref)	—
Thursday	1.00 (0.87 to 1.15)	0.97
Friday	0.85 (0.74 to 0.98)	0.03
Weekend admission*	0.90 (0.83 to 0.98)	0.01
Fifth of deprivation:		
1 (least deprived)	1.0 (ref)	—
2	0.98 (0.88 to 1.09)	0.68
3	0.92 (0.82 to 1.03)	0.13
4	0.82 (0.72 to 0.92)	0.001
5 (most deprived)	0.76 (0.66 to 0.88)	<0.001
Date of surgery (to most recent, increasing in 6 month intervals)	1.13 (1.10 to 1.15)	<0.001
Fracture volume of hospital (increasing in tenths)	1.02 (0.97 to 1.08)	0.46

*Defined as Friday or Saturday admission, as surgery most commonly occurs on day after admission. “Weekday” was therefore defined as Sunday-Thursday. “Weekend” included in logistic model as dichotomous variable and day of week excluded as collinear.

We conducted a further analysis among individuals with a non-pathological displaced intracapsular fracture who did not fulfil the NICE eligibility criteria for THA (n=92 926). Of these patients, 4903 underwent the procedure. With the same regression model, similar inequalities emerged. The receipt of THA outside the recommendations of NICE was least common among those with worse socioeconomic deprivation (odds ratio 0.64, 95% confidence interval 0.55 to 0.77), with a stepwise decrease from the most deprived fifth. Similarly, patients were less likely to receive THA outside the NICE guidelines when they were admitted at the weekend (0.89 (0.81 to 0.98) for all weekend, 0.87 (0.75 to 1.01) for Friday, 0.94 (0.81 to 1.09) for Saturday, 0.98 (0.84 to 1.15) for Sunday, 1.03 (0.89 to 1.19) for Monday, 1.01 (0.88 to 1.17) for Tuesday, 1.0 (reference) for Wednesday, and 1.01 (0.88 to 1.17) for Thursday.

## Discussion

This observational study used a large national audit dataset and has shown that there is unexplained variation in the use of THA after a hip fracture. This surgery was influenced by several characteristics of patients, including age, sex, cognition (AMTS), physical status (ASA score), socioeconomic status, and mobility before the fracture. Other key determinants were the treating hospital and the day of the week of admission. The use of THA among eligible patients increased over the study period but remains both low and variable.

### Compliance with NICE recommendations

NICE was established in 1999 to promote evidence based treatments and reduce unexplained variation in care across the NHS, the so called “postcode lottery.”^18^ In June 2011, NICE recommended that THA should be offered to patients with a displaced intracapsular hip fracture who can walk independently outdoors (with no more than a single mobility aid), are cognitively intact, and are medically fit to undergo the operation. This guideline is consistent with a developing evidence base, which suggests that THA leads to better functional outcomes than hemiarthroplasty after hip fracture,^7^ ^9^ ^10^ ^11^ although a large scale intervention study is needed and is currently underway.^19^ Despite the NICE guideline, we found that variation in the use of THA persists across the NHS because of poor compliance with the guidelines. There was substantial variation in compliance (0.1-60%) between hospitals. As patient level predictors were unable to account for this variation, it is likely to reflect systematic differences in practice between centres.

The optimal recursive partitioning model suggested that surgeons might consider factors that could be relevant even if not strictly included within the NICE guidelines. For example, older patients were less likely to undergo THA, as were those who mobilised using a stick compared with those mobilising independently without aids. Although there is strong evidence that some patients with hip fracture benefit from THA,^7^ ^9^ ^10^ ^11^ its precise indications are not well defined. Our model offers a glimpse into the collective judgment of orthopaedic surgeons and could be used to help inform the development of future NICE guidelines in the absence of higher level evidence. It is nevertheless concerning that deprivation was inversely associated with the use of THA. This observation persisted among patients who received a THA but did not meet the NICE guidelines, with deprived individuals least likely to inappropriately receive a THA. This is particularly important because NHS treatment is universally provided irrespective of ability to pay and “free at the point of use.” Challenging health inequalities is an ambition of initiatives aimed at increasing access to healthcare in other countries.^20^ It is therefore important to understand reasons for socioeconomic inequalities that persist in public healthcare systems. There are many potential explanations for this observation, including patients’ preferences and confounding factors. It is also possible, however, that heuristic judgments about which patients are sufficiently “independent” to benefit from THA could be influenced by implicit surgeon bias. Social class biases have been shown to influence treatment decisions across a range of settings^21^ ^22^ ^23^ and could raise a barrier for patients who are otherwise eligible to undergo THA. This inverse association risks exacerbating health inequalities and is a further reason to promote clear, evidence based, national guidelines.

### Barriers to increased provision of THA 

One potential obstacle to delivering THA for all eligible patients with hip fracture is the availability of experienced hip surgeons. It is widely accepted that patients undergoing elective THA by a low volume surgeon have greater risks of dislocation, need for revision surgery, postoperative complications, and death.^24^ ^25^ ^26^ ^27^ ^28^ For this reason, many orthopaedic surgeons do not perform THA for hip fracture if this operation is not part of their routine elective practice. The limited availability of suitably experienced hip surgeons might account for the reduced use of this procedure observed at weekends. This finding is important in the context of recent proposals to introduce seven day services across the NHS.^29^ Although this discussion is principally framed around increased weekend mortality,^30^ ^31^ timely access to THA for fracture might also need to be examined. Regionalisation of hip fracture services seems a plausible means of ensuring equal access to THA, by enabling specialist hip surgeons to support such a service every day. Dedicated hip fracture centres have already been successfully piloted in Germany.^32^ ^33^ The potential benefits of regionalisation, however, would need to be weighed against competing considerations such as the desire of older adults to be treated close to their homes.

### Strengths and limitations of study

The main strength of this study was its use of a dataset that captures almost every patient with hip fracture (>95%) treated in England, Wales, and Northern Ireland. There were variables that aligned closely with the NICE eligibility criteria, which permitted the recommended treatment algorithm to be mapped over the administrative data recorded within the NHFD.

The principal limitation was that the NHFD does not record individual patient comorbidities and so it was not possible to determine if specific comorbid diseases were associated with differences in the use of THA. Some of the variables in our analysis (such as age and deprivation) could simply represent a tendency towards a greater burden of comorbidity. The American Society of Anesthesiologists (ASA) score, however, has been shown to have equivalent or even greater predictive value for mortality and complications than standard comorbidity measures, such as the Charlson comorbidity index).^34^ ^35^ ^36^ It is unlikely that patients assigned a score ≤2 (2=“mild systemic disease”) were medically unfit to undergo THA. The NHFD also does not include sufficient detail to understand clinical decision making at the individual patient level. For example, it is possible that THA was discussed with some patients and hemiarthroplasty was chosen after a balanced discussion of risk and benefit. The variation between hospitals in compliance with NICE guidelines, however, suggests that there is likely to be systematic differences with provision of THA.

### Conclusion

Compliance with the NICE guidance on THA for hip fracture seems poor, with many apparently eligible patients not undergoing the procedure. There continues to be substantial variation in practice between hospitals, which is not readily explained by differences at the patient level. The limited use of THA among patients from deprived areas, the inappropriately high use among patients from more affluent areas, and inequalities in the provision of treatment at the weekend are particular concerns. Despite clear national guidelines, it seems most likely that there are systematic differences with use of THA in hip fractures within this dataset. There have been substantial improvements in all of the quality indicators measured by the NHFD since its creation in 2004.^3^ The NHFD should consider reporting data on THA provision at the hospital level to help achieve greater consistency across the NHS.

**What is already known on this topic**

A defined subset of patients with hip fracture achieve better functional outcomes with total hip arthroplasty (THA) than with hemiarthroplasty
NICE guidelines indicate which patients should be offered THA

**What this study adds**

Compliance with NICE guidelines is poor, and there is considerable variation between hospitals
Surgeons seem to apply different eligibility criteria than NICE
Socioeconomic deprivation and need for hip fracture surgery at the weekend are particular barriers to use of THA
Further efforts are necessary to improve the use of THA for eligible patients and reduce unexplained variation in care for older adults with hip fractures

## Web extra

Extra material supplied by author

Appendix 1: Mobility scores

Appendix 2: Hospitals contributing to database

## Notes

Ethical approval: The study protocol was approved by the Healthcare Quality Improvement Partnership (HQIP) before data release, but research ethics committee approval was not sought for secondary analysis of administrative data in line with Governance Arrangements for Research Ethics Committee (GAfREC) guidelines

Data sharing: Pursuant to the terms of our data sharing agreement with the National Hip Fracture Database no additional data can be made available by the authors.

Transparency: DP (the manuscript’s guarantor) affirms that the manuscript is an honest, accurate, and transparent account of the study being reported; that no important aspects of the study have been omitted; and that any discrepancies from the study as planned (and, if relevant, registered) have been explained.
