# Supplementary material for: Inequalities in use of total hip arthroplasty for hip fracture: population based study
Source: BMJ. 2016 Apr 28;353:i2021. doi: 10.1136/bmj.i2021 (PMC4849171; doi:10.1136/bmj.i2021)
Supplement: Supplementary file 1 — Appendix 1: Mobility scores [file perd029647.ww1_default.pdf]

Appendix 1: Mobility Scores [posted as supplied by author]

Version 7 and 8 of NHFD have different mobility scores

V7 therefore tracked onto V8

MOBILITY (VERSION 7 ONLY)

WalkInside		Walking Ability Indoors **(V7 only)**
	0	Regulary walked without aids
	1	Regularly walked with one aid
	2	Regularly walked with two aids or frame
	3	Wheelchair or bedbound
	4	Unknown
.		Missing

WalkOutside		Walking ability outside **(V7 only)**
	0	Regulary walked without aids
	1	Regularly walked with one aid
	2	Regularly walked with two aids or frame
	3	Wheelchair or bedbound
	4	Never goes outdoors
	5	Electric buggy
	6	Unknown
.		Missing

AccompOutside		Accompanied to Walk Ouside **(V7 only)**
	0	No
	1	Yes
	2	Wheelchair or bedbound
	3	Unknown
	4	Never goes outdoors
.		Missing

AccompInside		Accompanied to Walk Inside **(V7 only)**
	0	No
	1	Yes
	2	Wheelchair or bedbound
	3	Unknown
.		Missing

MOBILITY VERSION 8 ONLY

**Mobility**		**Pre-Fracture Walking Ability (VERSION 8 ONLY)**
	1	Freely mobile without aids
	2	Mobile outdoors with one aid
	3	Mobile outdoors with two aids or frame

4 Some indoor mobility but never goes outside without help
5 Unknown
6 No functional mobility

V7 TRACKED ONTO V8
(variable name = mob)

1	Freely mobile without aids (walkoutside 0)	
2	Mobile outdoors with one aid (walkoutside 1)	
3	Mobile outdoors with two aids or frame (walkoutside 2)	
4	Some indoor mobility but never goes outside without help	ANY WALKING INSIDE(0-2) AND ACCOMPANIEDOUTSIDE (1,2 and 4) OR ANYWALKING INSIDE(0-2) AND WALKINGOUTDOORS WHEEL/ BUGGY (3, 4,5)
5	No functional ability	Wheelchair or bedbound inside (walkinside 3)
6	Unknown	

STATA CODE

```
replace mob=mobility

replace mob=5 if walkinside==3

replace mob=4 if (walkinside==0 & accompoutside==1)

replace mob=4 if (walkinside==1 & accompoutside==1)

replace mob=4 if (walkinside==2 & accompoutside==1)

replace mob=4 if (walkinside==0 & accompoutside==2)

replace mob=4 if (walkinside==1 & accompoutside==2)

replace mob=4 if (walkinside==2 & accompoutside==2)

replace mob=4 if (walkinside==0 & accompoutside==4)

replace mob=4 if (walkinside==1 & accompoutside==4)

replace mob=4 if (walkinside==2 & accompoutside==4)

replace mob=4 if (walkinside==0 & walkoutside==3)

replace mob=4 if (walkinside==1 & walkoutside==3)

replace mob=4 if (walkinside==2 & walkoutside==3)

replace mob=4 if (walkinside==0 & walkoutside==4)
```

```
replace mob=4 if (walkinside==1 & walkoutside==4)
replace mob=4 if (walkinside==2 & walkoutside==4)
replace mob=4 if (walkinside==0 & walkoutside==5)
replace mob=4 if (walkinside==1 & walkoutside==5)
replace mob=4 if (walkinside==2 & walkoutside==5)
replace mob=3 if walkoutside==2
replace mob=2 if walkoutside==1
replace mob=1 if walkoutside==0
```
