# Supplementary material for: Inequalities in use of total hip arthroplasty for hip fracture: population based study
Source: BMJ. 2016 Apr 28;353:i2021. doi: 10.1136/bmj.i2021 (PMC4849171; doi:10.1136/bmj.i2021)
Supplement: Supplementary file 2 — Appendix 2: Hospitals contributing to database [file perd029647.ww2_default.pdf]

Appendix 2: Hospitals contributing to database [posted as supplied by authors]

Group 'X' Hospitals

ADD. Addenbrookes Hospital
AEI. Royal Albert Edward Infirmary
AIR. Airedale General Hospital
ASH. Wansbeck General Hospital
BAR. Barnsley District General Hospital
BAS. Basildon Hospital
BAT. Royal United Hospital Bath
BFH. Broomfield Chelmsford
BOL. Royal Bolton Hospital
BRD. Bradford Royal Infirmary
BRG. Bronglais General Hospital
BRI. Bristol Royal Infirmary
CCH. Charing Cross Hospital
CHE. Chesterfield Royal
CHG. Cheltenham General Hospital
COC. Countess of Chester Hospital
CRG. Craigavon Area Hospital
DAR. Darlington Memorial Hospital
DGE. Eastbourne DGH
DVH. Darent Valley Hospital
EAL. Ealing Hospital
EBH. Birmingham Heartlands Hospital
ESU. East Surrey Hospital
FAZ. University Hospital Aintree
FGH. Furness General
FRM. Frimley Park Hospital
FRY. Frenchay Hospital
GGH. Diana Princess of Wales Hospital
GHS. Good Hope General Hospital
GLO. Gloucestershire Royal Hospital
GRA. Grantham And District General Hospital
GWY. Ysbyty Gwynedd Hospital
HAR. Harrogate District Hospital
HCH. County Hospital Hereford
HIN. Hinchingbrooke Hospital
HOM. Homerton Hospital
HRI. Hull Royal Infirmary
IOW. St Marys Hospital Newport
JPH. James Paget Hospital

LDH. Luton & Dunstable Hospital
LGH. Leighton Hospital
LGI. Leeds General Infirmary
LIN. Lincoln County Hospital
LON. Royal London Hospital
MAC. Macclesfield District General Hospital
MKH. Milton Keynes General Hospital
MOR. Morriston Hospital
MPH. Taunton & Somerset Hospital
NCR. New Cross Hospital
NEV. Nevill Hall Hospital
NGS. Northern General Hospital
NHH. North Hampshire Hospital
NMG. North Manchester General Hospital
NMH. North Middlesex Hospital
NTG. University Hospital of North Tees
NTH. Northampton General Hospital
NUH. Ulster Hospital
OHM. Royal Oldham Hospital
PAH. Princess Alexandra Hospital
PIN. Pinderfields General Hospital
PLY. Derriford Hospital
PMS. The Great Western Hospital
QAP. Queen Alexandra Hospital
QEB. Queen Elizabeth Hospital Edgbaston
QEG. Queen Elizabeth Hospital Gateshead
QKL. Queen Elizabeth Hospital (Kings Lynn)
RAD. John Radcliffe Hospital
RBE. Royal Berkshire Hospital
RCH. Royal Cornwall Hospital
RDE. Royal Devon & Exeter Hospital
RED. The Alexandra Hospital
RFH. Royal Free Hospital
RGH. Royal Glamorgan
RHC. Royal Hampshire County Hospital
RLI. Royal Lancaster Infirmary
RLU. Royal Liverpool University Hospital
ROT. Rotherham General Hospital
RPH. Royal Preston Hospital
RSC. Royal Sussex County Hospital
RSS. Royal Shrewsbury Hospital
RSU. Royal Surrey County Hospital
RVB. Royal Victoria Hospital
RVN. Royal Victoria Infirmary
SAL. Salisbury District Hospital
SAN. Sandwell District Hospital

SCA. Scarborough General Hospital
SCM. James Cook University Hospital
SDG. Staffordshire General Hospital
SEH. Southend Hospital
SGH. Southampton General Hospital
SHH. Stepping Hill Hospital
SLF. Hope Hospital
SMV. Stoke Mandeville Hospital
SOU. Southport and Formby District General
SPH. St Peters Hospital
STD. South Tyneside District Hospital
STH. St Thomas Hospital
STM. St Marys Hospital Paddington
STO. University Hospital of North Staffordshire
STR. St Richards Hospital
SUN. Sunderland Royal Hospital
TOR. Torbay Hospital
TRA. Trafford General Hospital
TUN. Tunbridge Wells Hospital
UCL. University College Hospital
UHC. University Hospital Coventry
UHW. University Hospital of Wales
WAR. Warwick Hospital
WDG. Warrington District General Hospital
WES. Chelsea & Westminister Hospital
WEX. Wexham Park Hospital
WGH. Weston General Hospital
WHC. Whipps Cross Hospital
WHH. William Harvey Hospital
WHI. Whiston Hospital
WIR. Arrowe Park Hospital
WRG. Worthing Hospital
WRX. Maelor Hospital
WSH. West Suffolk Hospital
WWG. West Wales General
YDH. York District Hospital
YEO. Yeovil District Hospital
ENH. East and North Herts Hospital
QEW. Queen Elizabeth II Hospital (Welwyn)

Group 'Y' Hospitals

ALT. Altnagelvin Hospital
BED. Bedford Hospital
BLA. Royal Blackburn Hospital
BNT. Barnet General Hospital
BRO. Princess Royal University Hospital (Bromley)
BRT. Queens Hospital
BRY. Fairfield General Hospital
BSL. Bassetlaw District General Hospital
CHS. Chase Farm Hospital
CLW. Glan Clwyd DGH Trust
CMI. Cumberland Infirmary
COL. Colchester General Hospital
DER. Royal Derby Hospital
DID. Doncaster Royal Infirmary
DRY. University Hospital of North Durham
GEO. St Georges Hospital
GWE. Royal Gwent Hospital
GWH. Queen Elizabeth Hospital Woolwich
HIL. Hillingdon Hospital
HOR. Horton General Hospital
HUD. Huddersfield Royal Infirmary
IPS. The Ipswich Hospital
KCH. Kings College Hospital
KGH. Kettering General Hospital
KMH. Kings Mill Hospital
KTH. Kingston Hospital
LER. Leicester Royal Infirmary
LEW. University Hospital Lewisham
MAY. Mayday University Hospital
MDW. Medway Maritime Hospital
MRI. Manchester Royal Infirmary
NDD. North Devon District Hospital
NOB. Nobles Hospital
NOR. Norfolk and Norwich Hospital
NPH. Northwick Park Hospital
NTY. North Tyneside Hospital
NUN. George Eliot Hospital
NWG. Newham General Hospital
OLD. Queens Hospital Romford
PCH. Prince Charles Hospital
PET. Peterborough City Hospital
PGH. Poole General Hospital
PIL. Pilgrim Hospital

POW. Princess Of Wales Hospital
QEQ. Queen Elizabeth the Queen Mother Hospital
RUS. Russells Hall Hospital
SCU. Scunthorpe General Hospital
SHC. St Helier Hospital
TGA. Tameside General Hospital
TLF. Princess Royal Hospital Telford
UHN. University Hospital Queens Medical Centre
VIC. Victoria Hospital
WAT. Watford General Hospital
WDH. Dorset County Hospital
WHT. Whittington Hospital
WMH. Manor Hospital
WMU. West Middlesex University Hospital
WRC. Worcestershire Royal Hospital
WYB. Withybush General Hospital
WYT. Wythenshawe Hospital
MAI. Maidstone General Hospital

Hospitals not contributing to analysis owing to absent or insufficient data

KSX. Kent & Sussex Hospital
CGH. Conquest Hospital
CMH. Central Middlesex Hospital
DMO. Demonstration Medical Centre
PEH. Princess Elizabeth Hospital
SHJ. Jersey General Hospital
WCI. West Cumberland Infirmary
LIS. Lister Hospital
